# About Distress in Chronic Pain Conditions: A Pre–Post Study on the Effectiveness of a Mindfulness-Based Intervention for Fibromyalgia and Low Back Pain Patients

**DOI:** 10.3390/ijerph21111507

**Published:** 2024-11-13

**Authors:** Rebecca Ciacchini, Ciro Conversano, Graziella Orrù, Francesca Scafuto, Silvia Sabbatini, Mery Paroli, Mario Miniati, Alessio Matiz, Angelo Gemignani, Cristiano Crescentini

**Affiliations:** 1 School of Advanced Studies, University of Camerino, 62032 Camerino, Italy; 2 Department of Surgical, Medical, Molecular Pathology and Critical Care Medicine, University of Pisa, 56126 Pisa, Italy; ciro.conversano@unipi.it (C.C.); graziella.orru@unipi.it (G.O.); silviasabbatini.94@gmail.com (S.S.); angelo.gemignani@unipi.it (A.G.); 3 Department of Languages and Literatures, Communication, Education and Society, University of Udine, 33100 Udine, Italy; francescascafuto.80@gmail.com (F.S.); alessio.matiz@uniud.it (A.M.); cristiano.crescentini@uniud.it (C.C.); 4 Department of Clinical and Experimental Medicine, University of Pisa, 56126 Pisa, Italy; mery.paroli@gmail.com (M.P.); mario.miniati@med.unipi.it (M.M.); 5 Department of Psychology, Sapienza University of Rome, 00185 Rome, Italy

**Keywords:** stress, mindfulness, chronic pain, fibromyalgia, low back pain, meditation, distress, clinical psychology

## Abstract

Chronic pain (CP) affects about 30% of the global population and poses significant challenges to individuals and healthcare systems worldwide. The interactions between physiological, psychological, and social factors are crucial in the onset and development of CP conditions. This study aimed to evaluate the effectiveness of mindfulness-based intervention, examining its impact on perceived stress (PSS), depression and anxiety (BDI-II, PGWBI/DEP, SAS, STAI Y), sleep quality (PSQI), and mindfulness abilities (MAAS) in individuals with CP. Participants (N = 89, 84.3% female) underwent one of two diagnoses [fibromyalgia (FM) or low back pain (LBP)] and took part in an MBSR intervention. The mindfulness program proved effective in reducing PSQI scores (F = 11.97; *p* < 0.01) over time, independently of the type of diagnosis. There was also a marginal increase in trait mindfulness as measured by MAAS (F = 3.25; *p* = 0.07) in both groups. A significant difference between the two groups was found for the effect on PSS: F (1,87) = 6.46; *p* < 0.05. Mindfulness practice also reduced anxiety in FM and depressive symptoms in LBP, indicating a reduction in psychological distress among participants. Our findings suggest that mindfulness-based interventions may offer promising avenues for personalized pain management in clinical settings.

## 1. Introduction

Chronic pain (CP) is a complex and widespread issue, affecting around 30% of the global population [1,2]. There is a strong consensus that CP involves intricate interactions between physiological, psychological, and social factors [3,4]. Psychological variables significantly influence the management of CP [5,6,7,8,9], with research highlighting for instance how interoceptive traits like openness, emotional awareness, and self-regulation can enhance placebo-induced analgesia [10]. Recent studies suggest that CP should be viewed as a distinct condition shaped by genetic predispositions, neural adaptations, and environmental triggers. The present study considers two specific conditions of chronic pain: fibromyalgia (FM), characterized by widespread musculoskeletal pain, fatigue, and sleep disturbances, often accompanied by cognitive impairments and heightened sensitivity to pain, as well as low back pain (LBP), which refers to pain localized in the lower back that can result from various factors.

Neuroplasticity, stress perception, inflammatory pathways, and poor sleep quality are critical factors in maintaining pain states [11,12]. Psychological variables, including depression, anxiety, alexithymia, sexual health, and cognitive strategies, also significantly influence pain perception and processing in chronic conditions. Elevated levels of depression and anxiety can heighten the emotional distress associated with pain, particularly when comorbid with alexithymia. Additionally, sexual health issues may exacerbate the distress. Cognitive patterns, such as catastrophizing—where individuals expect or focus on the worst possible outcomes—can intensify pain perception. Conversely, cognitive strategies like reappraisal or distraction can either alleviate or worsen the pain experience, depending on their application [13,14,15,16,17,18,19]. Conversely, chronic pain (CP) may contribute to cognitive confusion, further complicating psychological well-being [20]. Neuroplastic processes in chronic pain conditions are often associated with central sensitization, where the central nervous system becomes more responsive to pain signals, leading to heightened sensitivity and the persistence of pain [21]. Furthermore, studies have revealed structural brain changes in CP, including reduced gray matter density and altered connectivity in key brain regions [22,23,24,25,26], with neuroplasticity sometimes becoming maladaptive, perpetuating pain [27].

Also, perceived stress plays a key role in CP, interacting with neurobiological pathways to influence pain experience and intensity [28,29,30]. Stress perception activates the hypothalamic–pituitary–adrenal (HPA) axis, releasing cortisol, which can modulate pain sensitivity and exacerbate CP [31]. Stress also affects neurotransmitter systems, releasing neuropeptides and altering glutamate signaling, which amplifies pain signals in the central nervous system [32,33].

Not only neuroinflammation but also all the processing of general body inflammation induced by the activation of the immune system contribute to the development and maintenance of some CP conditions [34,35,36]. While inflammation is a natural response, chronic inflammation contributes to persistent pain [37]. In CP, immune dysregulation leads to prolonged inflammation and increased sensitivity to pain signals; recent studies suggest targeting molecules like chemokines and cytokines to address neuroinflammation and pain processing [36,38,39].

CP also disrupts sleep, causing discomfort, muscle stiffness, and frequent awakenings, which worsen pain and reduce quality of life [40,41,42]. Anxiety and depression, often linked to CP, can contribute to insomnia and non-restorative sleep [43], while feelings of hopelessness and isolation exacerbate these conditions [44,45,46].

### 1.1. Mindfulness Interventions for Chronic Pain

Mindfulness refers to paying attention to one’s thoughts and feelings without judgment, being fully present in the moment, and cultivating a non-reactive awareness of experiences as they arise [47]. It is often developed through meditation practices (Vipassana, Samatha) and yoga, and has gained attention in scientific research for its potential benefits on mental and physical health [48]. Among non-pharmacological treatments, mindfulness-based approaches show promising results in the chronic pain (CP) field [49,50].

Mindfulness-based interventions (MBIs) come in various forms. The first, MBSR (mindfulness-based stress reduction), developed by Kabat-Zinn in 1979, specifically focuses on stress reduction [51,52]. Later, MBIs addressed other areas such as pain or addiction reduction, interpersonal communication, compassion, and support for pregnancy and parenthood [53]. Studies have shown that meditation practice is linked to structural and functional changes in the brain, such as increased gray matter density in areas related to learning, memory, and emotional regulation [54,55,56]. Even 8-week programs with meditation-naïve participants induce brain changes similar to those seen in long-term meditators [57]. Mindfulness meditation may also regulate immune responses and reduce pro-inflammatory cytokine production [58,59]. Research has demonstrated reductions in inflammatory biomarkers like C-reactive protein (CRP) in individuals with chronic inflammatory conditions following mindfulness-based interventions [60]. Numerous studies support the effectiveness of mindfulness-based protocols in addressing CP [61,62,63]. For instance, Anheyer et al. found short-term improvements in pain intensity and physical functioning in individuals with LBP after MBSR [64]. Similarly, patients with FM reported reduced stress levels, increased quality of life (QoL), and perceived well-being after MBSR participation [50,58,65,66,67].

Despite increasing evidence supporting mindfulness-based interventions (MBIs) like MBSR in managing CP, several gaps remain in the literature. There is limited research on the specific impact of MBSR across different CP conditions, for instance, comparing FM and LBP. Additionally, while the psychological benefits of mindfulness are well documented, further exploration of the clinical significance of these effects and long-term outcomes is needed, particularly within real-world settings like the Italian healthcare system, where access to comprehensive CP management remains inconsistent.

### 1.2. Research’s Rationale

Worldwide, CP seems to affect approximately 30% of the general population [1,68]. According to recent research spanning from 2019 to 2021, the prevalence of CP among U.S. adults peaked at 21.8%, with high-impact CP accounting for 7.8% [69]. In Italy, CP affects almost 28% of the population, which is higher than the average European prevalence; this higher percentage may be explained by the lack of consistent treatment options for CP in Italy, where nearly 68% of patients report inadequate access to comprehensive care [70]. In Italy, current treatment guidelines for CP emphasize a multidisciplinary approach that combines both pharmacological and non-pharmacological interventions. Pharmacological treatments typically include analgesics; nonsteroidal anti-inflammatory drugs (NSAIDs); and, in more severe cases, opioids or antidepressants. Non-pharmacological strategies recommended in the guidelines include physical therapy; cognitive behavioral therapy (CBT); and complementary approaches such as mindfulness-based stress reduction (MBSR) and other mindfulness-based interventions (MBIs). Despite these recommendations, access to comprehensive pain management programs remains limited in many regions, underscoring the need for integrating evidence-based approaches like mindfulness into the broader treatment landscape for chronic pain in Italy.

## 2. Materials and Methods

The present research was approved by the Hospital Ethical Committee (CEAVNO, Prot n. 16323 and Prot n. 19149).

### 2.1. Aim

Our aim was to propose and evaluate the effectiveness of a mindfulness-based intervention, specifically MBSR, assessing its impact on psychological variables such as sleep quality and mindfulness abilities and in reducing perceived stress in individuals with CP (FM and LBP patients). The study period included in this research was from September 2019 to September 2023, but the project is still ongoing. The research was proposed and implemented at the University of Pisa and the Santa Chiara Hospital (Pisa, Tuscany, Italy). In addition to assessing the overall effectiveness of the mindfulness-based intervention, we also explored potential gender-related differences as secondary outcomes.

### 2.2. Study Design

Considered the pilot-testing of the program, the study design was a non-randomized longitudinal pre–post study without a control group. The intervention is the MBSR, an evidence-based program developed by Jon Kabat-Zinn in the late 1970s at the University of Massachusetts Medical Center [71]. It aims to cultivate mindfulness, which involves paying attention to the present moment in a non-judgmental manner. The structure of MBSR typically consists of an 8-week course, with sessions lasting around 2.5 h each week. The program integrates various practices, including mindfulness meditation, body scan, yoga, and group inquiries. Participants are encouraged to practice the learned techniques both formally and informally in their daily lives. Through these practices, MBSR aims to help individuals develop greater awareness, acceptance, and resilience in the face of stress, pain, and other challenges.

### 2.3. Participants

Participants in the present study (N = 89) were patients referred to two different clinics within the Santa Chiara Hospital in Pisa, Italy. The clinics involved are the Rheumatology Unit for patients with FM (n = 49) and the Anaesthesiology and Pain Therapy Unit for patients with LBP (n = 40). All patients were diagnosed with CP (either FM or LBP). All of them received standard pharmacological treatment at their pain unit before, during, and after the intervention (i.e., pain relievers, antidepressants, anticonvulsants, muscle relaxants, etc.). Due to limited access to medical records, we were unable to verify specific treatments, thus enabling a broader range of participants to join the intervention.

### 2.4. Procedures

During a routine appointment, the healthcare professional inquired whether the patient would like to take part in a study, briefly explaining the aim of investigating the impacts of meditation on CP. If the patient expressed interest, arrangements were made for them to meet with both the MBSR mindfulness meditation teacher and the study researchers. The inclusion criteria were as follows: age between 18 and 85 years, signed informed consent, diagnosis of CP from LBP or FM, and a good understanding of the Italian language. The exclusion criteria were as follows: severe acquired neurological disorders, psychiatric disorders inadequately managed by pharmacological therapy, documented cognitive deficits, pregnancy status at the time of recruitment, and orthostatic hypotension. Following the selection criteria, instructors and researchers selected eligible patients for the study, who were then contacted via email or phone to inform them about the start of the mindfulness course, including its dates, timing, and schedule. As previously mentioned, the program adhered to was MBSR, comprising eight weekly sessions dedicated to group-based formal and informal meditation practices. The MBSR intervention followed the standard 8-week program structure. However, the movement-based yoga component was not included for the LBP group due to physical limitations, whereas participants in the FM group were given the option to perform the yoga practices while seated, if needed. Participants were allowed to miss a maximum of one session out of the eight-week program, ensuring that all participants attended at least seven sessions. Attendance was particularly emphasized for the 4th and 5th sessions, which were deemed crucial for stress management and emotional regulation. During the research period, five MBSR groups for patients with FM and four for patients with LBP were administrated, each comprising approximately 10 individuals. The choice of targeting a smaller group was made to provide the most effective intervention possible and to minimize the time between recruitment and the start of the group to maintain patients’ interest. As per the measurement method, a preliminary introductory meeting was organized, as expected by the MBSR program, during which practical information on navigating the course was provided to participants. At this juncture, psychometric measurements were collected (pre-intervention measures), with a response rate of 100% from the participants. At the conclusion of the final meeting, the eighth one, the same questionnaires were administered (post-intervention measures). All 89 participants completed the post-intervention measurements, except for the demographic questionnaire, which was omitted.

### 2.5. Measures

This research focused on two main areas: (a) psychological variables impacting well-being and quality of life, such as perceived stress and sleep quality, and (b) the presence or absence of mindfulness attitudes. Below is a list of the measures used.

(a) Sociodemographic aspects
  - Ad hoc questionnaire.
(b) Questionnaires for measuring psychological variables
  - Perceived Stress Scale (PSS) [72];
  - Pittsburgh Sleep Quality Index (PSQI) for sleep quality [73];
  - Beck Depression Inventory-II (BDI-II) (LBP groups) [74] and Psychological General Well-Being Index (PGWBI) subscale Depression (DEP) to evaluate depression levels (FM groups) [75];
  - Self-Rating Anxiety Scale (SAS; FM groups) [76] and State–Trait Anxiety Inventory—Form Y (STAI-Y; LBP groups) [77] for anxiety assessment.
(c) Presence/absence/improvement of mindfulness attitudes
  - Mindful Attention Awareness Scale (MAAS) [78].

### 2.6. Measures Descriptions:

1. An ad hoc questionnaire was designed to collect demographic data from participants as, for example, age and gender (only before the intervention);
2. Perceived Stress Scale (PSS-10) [72]: The PSS-10 is a widely recognized self-report tool designed to gauge an individual’s perception of stress. Participants are asked to rate their responses on a Likert scale ranging from 0 to 4 (0 = never; 1 = almost never; 2 = sometimes; 3 = quite often; 4 = very often). While most questions aim to capture negative feelings and challenges associated with stress, a subset of inquiries explores positive emotions and coping mechanisms in stressful situations. PSS-10 scores can range from 0 to 40, with higher scores indicative of elevated levels of perceived stress.
3. Pittsburgh Sleep Quality Index (PSQI) [73]: The PSQI is a standardized questionnaire designed for self-administration, assessing retrospective sleep quality and disturbances experienced in the past month. Comprising 19 items, the questionnaire is divided into 7 subscales: (1) sleep quality (1 item), (2) sleep latency (2 items), (3) sleep duration (1 item), (4) sleep efficiency (3 items), (5) sleep disturbance (9 items), (6) sleep medication (1 item), and (7) daily dysfunction (2 items). Each item is scored on a scale ranging from 0 to 3. The sum of scores across these seven components produces a PSQI global score, which can range from 0 to 21. Individuals with a global score exceeding 5 are categorized as ‘poor sleepers’.
4. Beck Depression Inventory-II (BDI-II) [74]: The BDI-II is composed of 21 items aimed at evaluating the severity of depression. Each item consists of a set of four statements organized from least to most severe regarding a specific depression symptom, with respondents assigning a score ranging from ‘0′ (absent) to ‘3′ (strongly present). Consequently, total scores can range from 0 to 63. Respondents are asked to consider the preceding 2 weeks when responding. Guidelines for severity ratings and suggested cutoff scores for individuals diagnosed with major depression are as follows: 0–13 = ‘minimal’; 14–19 = ‘mild’; 20–28 = ‘moderate’; and 29–63 = ‘severe’. This measure was used in the group with a diagnosis of LBP.
5. Psychological General Well-Being Index (PGWBI) [75]: This is a self-reported questionnaire used to assess an individual’s psychological well-being across six subscales. The PGWBI consists of 22 items designed to measure psychological well-being across 6 dimensions, namely anxiety; positive well-being; self-control; general health; vitality; and the subscale we considered for this study, which is depressed mood (DEP). Each item is rated on a 6-point scale, with responses ranging from “none of the time” to “all of the time”.
6. Self-Rating Anxiety Scale (SAS) [76]: The Self-Rating Anxiety Scale (SAS) is a 20-item assessment designed to evaluate the frequency of anxiety symptoms based on diagnostic frameworks. It primarily focuses on somatic symptoms. Respondents rate how frequently they have experienced each symptom using a 4-point Likert scale: ‘‘none or a little of the time’’ (coded as 1), ‘‘some of the time’’ (coded as 2), ‘‘good part of the time’’ (coded as 3), and ‘‘most or all of the time’’ (coded as 4). Items 5, 9, 13, 17, and 19 are reverse-scored. Total scores on the SAS range from 0 to 80. This measure was used in the group with FM.
7. State–Trait Anxiety Inventory—Form Y [77]: The State–Trait Anxiety Inventory (STAI) is used to measure anxiety levels in individuals. There are two versions of the STAI: Form X and Form Y. Form Y (STAI-Y) consists of 40 items designed to assess both state anxiety (anxiety about an event or situation) and trait anxiety (general anxiety level). The items are rated on a 4-point scale, with responses ranging from “almost never” to “almost always”. The STAI-Y has been widely used in both clinical and research settings to assess anxiety levels across various populations. This measure was used just in the group with LBP.
8. Mindful Attention Awareness Scale (MAAS) [78]: The MAAS comprises 15 statements aimed at assessing a key aspect of mindfulness, characterized by an open and receptive cognitive tendency. This ability fosters a heightened awareness of the present moment, enabling individuals to observe ongoing events without excessive judgment. Participants are asked to reflect on their daily experiences and rate the frequency of each encounter on a scale ranging from 1 to 6. The total scale score is calculated by averaging responses to all 15 statements.

### 2.7. Data Analysis

The statistical analyses conducted in this study involved several procedures. First, we considered the total sample and the common outcome measures used for both subgroups (MAAS, PSQI, and PSS-10). We calculated descriptive statistics and analyzed the equivalence of means for the two subsamples with FM and LBP at pre-test (ANOVA for independent samples) to verify the equivalence of the two groups at pre-test. Consequently, we conducted MANOVA for repeated measures, estimating the main and interactive effects of group by time on MAAS, PSS-10, and PSQI. Finally, we conducted analyses in the two subsamples to verify the effectiveness of the program on anxiety (SAS or STAI-Y) and depression measures (DEP or BDI-II). The choice of splitting the sample was driven by the availability of different measures used to assess psychological distress (anxiety and depression) in the two groups. Therefore, we employed additional MANOVA for repeated measures in a single-group design separately for participants with FM and participants with LBP, specifically for anxiety and depression variables.

## 3. Results

### 3.1. Demographics and Preliminary Pre-Test Analyses

The total sample was composed of 89 participants: 49 with a diagnosis of FM and 40 with LBP. Most were women (84.3%) in a large range of age from 20 to 72, M = 48.04 (12.51). Preliminary analyses showed the equivalence of the groups at the pre-test on the outcome measures (PSS, PSQI, and MAAS). No significant differences were revealed in the pre-test, with PSS: F (1, 87) = 0.53, N.S.; PSQI: F (1, 87) = 0.83, N.S; and MAAS: F (1, 87) = 0.19, N.S.

Considering the amplitude of the age range and the prevalence of female gender, we also tested the effects of sociodemographic variables on the outcomes variables. No significant effect was revealed for gender on PSS: F (1, 87) = 1.5, N.S.; PSQI: F (1, 87) = 2.5, N.S; or MAAS: F (1, 87) = 0.03, N.S. Additionally, no significant correlation emerged between age and MAAS (r = 0.03), PSQI (r = −0.04), or PSS (r = −0.03) at the pre-test.

### 3.2. Changes in Stress, Sleep Quality, and Mindfulness

In order to verify the effectiveness of the program on the psychological variables mentioned above, common to both groups, MANOVA for repeated measures was performed with a between factor (Group: FM and LBP) and a within factor (time: pre-test and post-test) in the total sample (N = 89). The multivariate test showed the significant main effect of time (F (3, 85) = 13.75; *p* < 0.001), while the interaction effect of time X group was not significant: F (3, 85); *p* = 0.08 (Table 1).

The mindfulness program proved effective in reducing PSQI scores (F = 11.97; *p* < 0.01) over time, independently of the type of diagnosis (Table 1). The program did not significantly increase MAAS scores (dispositional mindfulness); however, a positive trend of improvement was revealed after the intervention (F = 3.25; *p* = 0.07) in both groups. Univariates showed that the only significant difference of means between the two groups was on the effect of PSS: F (1, 87) = 6.46; *p* < 0.05 (Table 1).

Thus, in both groups (FM and LBP), the mindfulness intervention showed a significant improvement in perceived stress (PSS-10) and sleep quality (PSQI) from the pre-test to the post-test. Specifically, the mean PSS-10 scores decreased from 24.00 to 18.45 in the FM group and from 22.95 to 20.55 in the LBP group. Regarding sleep quality, the mean PSQI scores decreased from 10.63 to 9.26 in the FM group and from 9.87 to 8.90 in the LBP group. For MAAS, both groups showed a slight increase, with scores increasing from 59.08 to 62.22 in the FM sample and from 57.90 to 60.05 in the LBP; however, this change did not reach statistical significance.

### 3.3. Anxiety and Depression

In order to verify the effectiveness of the program on psychological distress (anxiety and depression levels), the differences in means with MANOVA for repeated measures separately in the two subsamples of FM and LBP, with a single-group design, were analyzed. In the FM sample, the multivariate test showed a significant effect of the intervention at post-test (F (2, 47) = 11.68; *p* < 0.001). Univariates showed a significant effect on the self-rated anxiety score (SAS) and a trend of decrease in depression (DEP) (Table 2). It is worth noting that the self-rated anxiety score reached almost the cutoff score (41) after the intervention, with just two points above.

Furthermore, we applied a new MANOVA for repeated measures in the subsample of subjects with LBP. The multivariate test showed a significant effect of the intervention (F = 4.54; *p* < 0.01), while the univariate test showed specifically that the effect was just significant regarding depression (BDI-II) but not anxiety (STAI Y1-Y2) (Table 3). Hence, the intervention reduced depression but did not affect trait and state anxiety in LBP patients. It is noticeable that the BDI-II score after the intervention reached a score that was between the range of low and moderate symptoms (19.50).

## 4. Discussion

The present study aimed to observe the effectiveness of an MBI on various psychosocial outcomes in individuals with CP, specifically targeting perceived stress, depression, anxiety, sleep quality, and mindfulness abilities. A sample of 89 participants (84.3% female) diagnosed with FM or LBP took part in an MBSR program and pre–post psychometric testing sessions.

The preliminary analyses aimed to assess the equivalence of the participant groups at the pre-test stage concerning the outcome measures, including stress (PSS-10), sleep quality (PSQI), and mindfulness ability (MAAS). The results indicated no significant differences between the groups at pre-test across all outcome measures, suggesting that the groups were comparable at baseline. Furthermore, the effects of sociodemographic variables such as gender and age on the psychological variables were examined. Neither gender nor age significantly influenced the psychological variables measured in this study at the pre-test stage, suggesting that psychological variables related to CP may not be inherently tied to gender-specific factors or may not be significantly influenced by chronological age in our sample. Nevertheless, these data need to be interpreted with caution due to the high prevalence of female participants in the current study [79,80].

The multivariate test revealed a significant main effect of time, indicating overall changes in psychological variables such as stress (PSS-10) and sleep quality (PSQI) across both groups over the duration of the program. This finding aligns with the notion of the intervention directly impacting the observed variables, thus corroborating existing evidence in the literature.

The results of our study thus indicate that the mindfulness program was effective in reducing PSQI scores over time, meaning an improved sleep quality, regardless of whether the participants had FM or LBP. Scientific studies consistently suggest that mindfulness meditation can lead to improvements in sleep quality. Several studies have demonstrated that regular mindfulness meditation practice is associated with decreased insomnia symptoms, reduced sleep disturbances, and improved overall sleep quality [81,82,83,84,85,86]. One mechanism through which mindfulness meditation may improve sleep quality is by reducing arousal and promoting relaxation; mindfulness techniques help individuals cultivate a state of present-moment awareness and non-judgmental acceptance, which can counteract the rumination and worry often associated with sleep difficulties [87]. Moreover, mindfulness practices have been shown to regulate the autonomic nervous system and decrease physiological arousal, which can contribute to a more restful sleep environment [88].

Additionally, our results showed a trend toward increasing scores on the MAAS in both groups, although this increase was not significant. Dispositional (or trait) measures like the MAAS are typically less sensitive to short-term changes compared to state measures, which assess mindfulness levels at a specific point in time. Consequently, while participants may experience improvements in mindfulness awareness throughout the MBSR program, these changes may not be fully captured by a trait measure like the MAAS, leading to potentially less pronounced or statistically non-significant results [89]. Also, there was no significant difference in the effect of the program between individuals with FM and those with LBP regarding MAAS scores.

However, when considering perceived stress (PSS-10), there was a significant difference between the two groups, with the intervention being more effective in reducing perceived stress in the FM group compared to the LBP group. The varying nature and severity of pain experienced in FM versus LBP may have influenced how individuals perceive and respond to mindfulness intervention; it is possible that mindfulness meditation may be particularly effective in addressing the multifaceted nature of stress in FM, whereas the focus on localized pain management in LBP may not fully address broader stress-related issues. Moreover, potential differences in treatment expectations and adherence between individuals with FM and LBP, which we did not evaluate, may also contribute to variations in the effectiveness of mindfulness meditation on stress reduction; factors such as motivation, belief in the effectiveness of the intervention, and willingness to engage in mindfulness practices regularly could influence outcomes [90,91]. Another factor to account for this difference is that many of the patients with LBP were unable to move, and thus they did not engage in movement-based yoga practice participating in sessions while lying down, and this may have contributed to reduced effects of the training on perceived stress levels in LBP patients. This would highlight the possible role of body movement and bottom-up practices such as body movement and expressive behavior in releasing stress overload [92,93,94]. Furthermore, in both groups, the stress scores remained in the moderate range even after the intervention, namely still above the established cutoff point of 14. Although the small size of our sample does not allow for a generalization of the results to all patients with CP, this finding highlights the crucial role of perceived stress and its persistence in CP, calling for the need for other complementary interventions [92,93,94].

Continuing with our findings, we should note that, within the FM sample, the multivariate test unveiled a notable impact of the intervention on anxiety and depression, which are closely linked to psychological distress, at the post-test stage. Further examination through univariate tests showed that this effect was particularly pronounced for self-rated anxiety scores (SASs), with a significant decrease observed. Additionally, there was a trend in depression (DEP), indicating a positive trajectory of an improvement in depression levels, although it did not reach statistical significance. This observation is noteworthy as it highlights the substantial reduction in self-rated anxiety scores following the intervention for FM patients, almost reaching the established cutoff point.

In the subsample of LBP, the univariate analyses showed a main significant effect for depression (BDI-II) but not for anxiety (STAI Y1-Y2). Of note is the observation that the post-intervention BDI-II scores fell within the range indicative of low–moderate symptom severity (19.5, just above the cutoff for moderate), further emphasizing the positive impact of the intervention on depression levels [95].

Even though it is known that meditation-based interventions such as MBSR can reduce anxiety and depression in patients with various chronic conditions, recent studies shed light on the same discrepancies between the effects on anxiety and depression in the two samples as observed in our study [50,96]. The discrepancy in the effects on depression and anxiety found in the current samples of FM and LBP patients may depend on a few factors. Firstly, the measures used in the two subgroups are different, tapping into different aspects and symptoms of depression and anxiety. Secondly, the FM group may have depression symptoms that may be more “resistant” to the intervention, as they may be linked to personality factors that are more stable over time and more difficult to address with a two-month intervention; in fact, while the connection between depression and FM is consistently demonstrated, the timing of this association remains a topic of debate [97]. With regard to anxiety, patients with LBP might have different cognitive manifestations compared to patients with FM; patients with FM might be more concerned with the future in general and with whether they will have the strength to face daily situations, while patients with LBP might be more anxious in relation to situational circumstances, such as the outcomes of exams or surgical interventions [98]. These possible differences could make the interaction of different anxiety levels with variables arising from meditation practice highly complex. Meditation has indeed proven useful in reducing worrying and rumination but may be less effective in ‘acute’ situations when one needs to approach stressful circumstances.

### Strengths and Limitations

This research presents several limitations. Firstly, since the study design is non-randomized, there may be inherent biases in the selection of participants or allocation to intervention groups, which could impact the validity of the results. Secondly, the absence of a control group for comparison limits the ability to draw firm conclusions about the effectiveness of the intervention. Thirdly, the lack of control over potential confounding variables, such as concurrent treatments or changes in lifestyle factors, could introduce bias and limit the ability to attribute changes solely to the intervention. Another limitation is the wide age range of participants (18–85 years). We acknowledge that the causes of chronic pain and the response to mindfulness interventions may vary significantly between younger adults and older individuals. However, the number of participants was not sufficient to allow for stratification by age, and in a hospital setting, it is often not ethical to limit age groups too strictly, as this could restrict access to treatment opportunities. Future studies with larger and more stratified samples could better explore these differences. Another limitation is represented by the fact that these studies were conducted during a period of time that included the COVID-19 pandemic, which could have influenced the results in some way. Upon reviewing the timing of the interventions, it is observed that, concerning the LBP groups, these started in 2020 as the project approval and submission lasted longer, while the FM groups started earlier (2018). Evidence from the literature highlight how COVID-19 pandemic has affected people’s mental health on a large scale [99,100,101,102]. Hence, it could be possible that patients with LBP who started the program later, during the COVID-19 pandemic, had more disadvantages than the FM group. Finally, using different psychometric questionnaires for depression and anxiety in the two subgroups represents a limitation because it introduces variability in the measurement tools, potentially affecting the comparability of results between the subgroups. Additionally, different questionnaires and qualitative interviews may capture different aspects of the constructs being measured, leading to inconsistencies in the data analysis and interpretation. Finally, while we assessed pain intensity using the Visual Analog Scale (VAS) in a subset of participants, the self-reported nature of the data, combined with issues in data collection for some individuals, prevented the inclusion of these results in the current analysis. As such, the impact of the intervention on pain experience and pain interference was not explored in this study. Future research with more robust pain assessments, potentially including qualitative analyses, can provide deeper insights into these aspects.

Despite these limitations, some strengths of the study should be underlined. This was a pilot study to verify the effectiveness and feasibility of an MBSR program in a context where no other interventions besides medication (neither psychotherapy nor complementary programs) were planned for CP patients. Generally speaking, the Public Italian Health System is still lacking psychological interventions to address CP and is far from applying guidelines that suggest the use of complementary and large-scale programs, together with psychotherapies. Thus, the intervention was innovative, considering the context of its application. A second strength of this study was feasibility since none of the patients dropped out. Hence, all of them completed the program, showing good acceptability. Nevertheless, we missed the qualitative reports to explore the meaning of their participation and the benefits they received. Hence, another added value was the specific adaptation of the program for LBP patients since they did not practice the yoga exercises.

## 5. Conclusions

These findings suggest that while the MBSR showed promise in improving sleep quality and increasing mindfulness awareness across both CP conditions, it had a more pronounced effect in reducing perceived stress, specifically in individuals with FM. The significant improvement in anxiety scores of FM individuals and depression levels of patients with LBP underscores the relevance of mindfulness interventions in addressing the emotional burden associated with CP conditions. While further investigation is needed to elucidate the specific characteristics in the intervention response among different pain conditions, our results suggest promising avenues for tailored pain-management meditation-based interventions in clinical practice.

## Figures and Tables

**Table 1:** Univariate Tests of Manova for repeated measures in the Total sample (N = 89).

Effect	Measures	F	*p*
Time	PSS	41.12	0.000
	PSQI	11.97	0.001
	MAAS	3.25	0.075
Time * GROUP	PSS	6.46	0.013
	PSQI	0.34	0.564
	MAAS	0.11	0.736

**Table 2:** Manova for repeated measures in the subsample of Fibromyalgia: estimated means and univariates for Anxiety (SAS) and Depression (DEP) (N = 49).

Measures	Time	Mean	S.E.	Confidence Interval 95%			
				Lower Limit	Upper Limit	F	*p*
SAS	Pre-test	48.61	1.50	45.60	51.63	22.04	0.000
	Post-test	42.71	1.65	39.40	46.02		
DEP	Pre-test	10.31	0.45	9.40	11.22	4.01	0.051
	Post-test	9.43	0.58	8.26	10.60		

**Table 3:** Manova for repeated measures in the subsample of Low Back Pain: estimated means and univariates for Anxiety (STAI-Y1; STAI-Y2) and Depression (BDI-II) (N = 40).

Measures	Time	Mean	S.E.	Confidence Interval 95%			
				Lower Limit	Upper Limit	F	*p*
STAI-Y1	Pre-test	47.07	2.01	43.00	51.14	0.019	0.891
	Post-test	47.25	1.83	43.55	50.95		
STAI-Y2	Pre-test	49.65	1.53	46.56	52.74	2.28	0.139
	Post-test	48.32	1.77	44.74	51.90		
BDI-II	Pre-test	22.75	1.61	19.49	26.00	10.29	0.003
	Post-test	19.50	1.58	16.30	22.70		

## Data Availability

The datasets presented in this article are not readily available because the data are part of an ongoing study. Requests to access the datasets should be directed to rebecca.ciacchini@med.unipi.it.
